# Tooth loss progression and mortality among older adults: results from the Chinese longitudinal healthy longevity survey (CLHLS)

**DOI:** 10.1186/s12877-025-06419-1

**Published:** 2025-10-10

**Authors:** Linjia Duan, Liu Yang, Haiyan Ruan, Halmira Alimjan, Liming Zhao, Ziqiong Wang, Lu Liu, Ningying Song, Sen He

**Affiliations:** 1 Department of Cardiology, West China Hospital, Sichuan University, Chengdu, China; 2 Department of Cardiology, Karamay Hospital of Integrated Chinese and Western Medicine, Karamay, China; 3 Department of Cardiology, Hospital of Traditional Chinese Medicine, Shuangliu District, Chengdu, China; 4 West China School of Nursing, Sichuan University, Chengdu, China; 5 Department of Cardiology, Hospital of Chengdu Office of People’s Government of Tibetan Autonomous Region, Chengdu, China; 6 Department of Otolaryngology‑Head & Neck Surgery, West China Hospital, Sichuan University, Chengdu, China

**Keywords:** Mortality, Older adults, Progression, Tooth loss

## Abstract

**Background:**

Previous studies on the association between tooth loss and mortality typically relied on a single assessment of baseline tooth count. Therefore, we aimed to examine whether tooth loss progression was associated with mortality independently of baseline tooth count among older adults, using data from the Chinese Longitudinal Healthy Longevity Survey.

**Methods:**

A total of 8073 older participants were included (median age: 83.0, interquartile range: 73.0–91.0; men: 46.6%). The exposure was tooth loss progression, which was assessed by annual tooth loss and categorized into four groups: stable (0 teeth/year), slow loss (> 0, < 2 teeth/year), moderate loss (≥ 2, < 4 teeth/year), and rapid loss (≥ 4 teeth/year). The study outcome was all-cause mortality, with Cox regression analysis used to examine the association between tooth loss progression and mortality.

**Results:**

During a median follow-up period of 3.5 years, a total of 5176 deaths (64.1%) were recorded. Overall, mortality risk significantly increased with a more rapid progression of tooth loss after adjusting for baseline tooth count and other confounding variables. Compared to the stable group, adjusted hazard ratios (HRs) for mortality were 1.11 (95% CI: 1.03–1.20) for the slow loss group, 1.20 (95% CI: 1.09–1.32) for the moderate loss group, and 1.33 (95% CI: 1.19–1.48) for the rapid loss group, respectively. Furthermore, in the restricted cubic spline models, a positive linear relationship was observed between annual tooth loss and mortality (adjusted HR: 1.04, 95% CI: 1.03–1.05, per one tooth loss).

**Conclusions:**

Among older adults, the risk of all-cause mortality significantly increased with a more rapid progression of tooth loss, regardless of baseline tooth count. These findings emphasize the critical importance of monitoring tooth loss progression.

**Supplementary Information:**

The online version contains supplementary material available at 10.1186/s12877-025-06419-1.

## Introduction

The Global Burden of Disease study has demonstrated that oral health represents a significant global population health challenge [1], and in November 2022, the World Health Organization released the Global Oral Health Status Report, which estimated that nearly 3.5 billion individuals worldwide (approximately 50% of the global population) suffer from various forms of oral disease [2]. Among these conditions, tooth loss stands out as one of the most prevalent issues. While tooth loss is frequently perceived as an inevitable consequence of aging [2–5], it can have profound psychological impacts, social repercussions, and is associated with a variety of adverse health outcomes [1, 2, 6–9]. Furthermore, tooth loss exhibits a high prevalence rate on a global scale [2, 3, 7, 10], with an estimated average prevalence for complete tooth loss approaching 7.0% in 2019 [2]. Therefore, tooth loss poses a substantial public health concern worldwide [1, 7].

Recently, emerging studies have revealed that tooth loss is significantly associated with an elevated risk of all-cause mortality [6, 8, 9, 11], as well as cause-specific mortalities including cardiovascular mortality [6, 9, 12], lung cancer mortality [11], upper gastrointestinal cancer mortality [12], and pneumonia mortality [13]. Despite the growing body of evidence linking tooth loss to these adverse outcomes, previous studies have predominantly relied on a single assessment of baseline tooth count. However, this approach may limit the robustness of observed associations since individuals can lose teeth throughout their lifespan [3, 14–16], particularly among older adults [3, 14]. Therefore, the strength of the association between tooth loss and mortality may vary over time; thus, evaluating tooth loss progression would provide a more comprehensive measure than a single evaluation.

To the best of our knowledge, there is a lack of comprehensive evaluation on the association between tooth loss progression and mortality among a substantial cohort of older adults. Furthermore, according to China’s seventh national census conducted in 2020, the country has a significant elderly population, with approximately 190.6 million individuals (13.5% of the total population) aged 65 years and older [17]. In this context, the present study aimed to investigate whether tooth loss progression was associated with mortality among older adults after adjusting for baseline tooth count, using data from the Chinese Longitudinal Healthy Longevity Survey (CLHLS). Given that certain factors influencing tooth loss progression may be modifiable [18, 19], this study holds potential significance for public health.

## Methods

### Study participants

The CLHLS is a nationwide, ongoing prospective cohort study of community-dwelling older adults in China. The primary goal of the study is to better understand the factors contributing to healthy longevity among Chinese adults aged 80 years and above, as well as those aged 65–79 years. The CLHLS was initiated in 1998 and has been updated at regular intervals since then (i.e., 2000, 2002, 2005, 2008, 2011, 2014, and 2018). To address attrition due to mortality and loss to follow-up, new participants have been enrolled since 2000. The survey was conducted in the residences of participants by well-trained interviewers, covering half of the counties and cities randomly selected from 23 provinces in China. The population within the survey areas represents approximately 85.0% of the total Chinese population. The CLHLS was approved by Peking University’s research ethics committee (IRB00001052–13074), and conducted in accordance with the principles outlined in the Declaration of Helsinki. Informed consent was obtained from all survey participants prior to their involvement. Further details about the CLHLS can be found in the published literature, which generally reports positive findings regarding data quality [20–22].

**Fig. 1:**
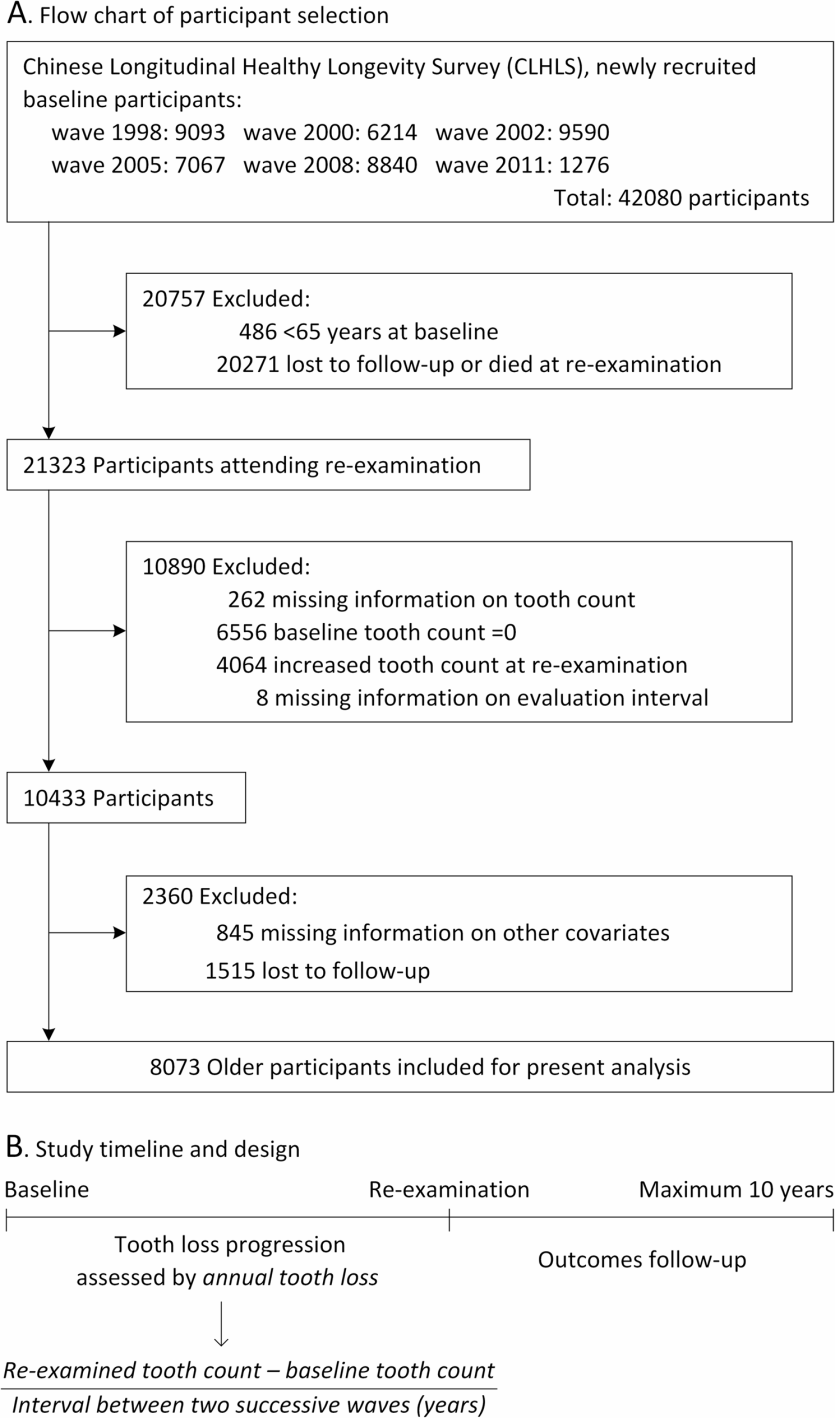
Flow chart of participant selection and study timeline

Figure 1A presents a detailed overview of participant selection. The inclusion criteria stipulated that participants must have undergone assessments of tooth count in two consecutive waves (e.g., 1998 and 2000, 2000 and 2002, etc.). Additionally, it was necessary for participants to have at least one tooth at baseline in order to facilitate the assessment of tooth loss progression during the re-examination. Furthermore, participants with tooth increase between two successive waves were excluded from the study, as it is less common for older adults to acquire new teeth. Ultimately, our study sample consisted of a total of 8073 older participants.

###  Assessment of tooth loss progression

Firstly, the number of teeth was assessed using the question “How many natural teeth do you have (excluding dentures)?” Subsequently, tooth loss progression was assessed based on examinations conducted over two successive waves. Assuming a constant rate of tooth loss over time, the progression was assessed using annual tooth loss to balance the interval between two successive waves, calculated as shown in Fig. 1B. Based on the distribution of annual tooth loss (eFigure 1) and subsequent cubic spline analysis, along with practical utility considerations, tooth loss progression was categorized into four groups: stable (0 teeth/year), slow loss (> 0 and < 2 teeth/year), moderate loss (≥ 2 and < 4 teeth/year), and rapid loss (≥ 4 teeth/year).

### Assessment of covariates

The detailed information on covariates is presented in eTable 1. This included data on sex (male/female), age, education (no school/1 year or more), marital status (in marriage/not in marriage), residence (urban/rural), co-residence (with family members/alone/in a nursing home), current smoking (yes/no), current drinking (yes/no), current regular exercise (yes/no), regular intake of foods (fruit, vegetable, meat, fish, eggs, and beans; yes/no), comorbidities (hypertension, diabetes, heart disease, cerebrovascular disease, and respiratory disease; yes/no), activities of daily living (ADL) disability (yes/no), and denture (yes/no). All covariates were self-reported, and the age of participants was further validated through the use of an identification card or household registration book. For a more comprehensive understanding of the details provided, please refer to: https://agingcenter.duke.edu/CLHLS.

### Study outcome

The study outcome was centered on all-cause mortality, defined as death resulting from any cause. Survival status and date of death were acquired through interviews with close relatives at each survey, and then, the information was validated using available medical documentation. The follow-up period was calculated from the re-examination to either the date of death, the last follow-up interview, or the censoring time (i.e., a 10-year period) for each participant, whichever occurred first (Fig. 1B). In the CLHLS dataset, mortality data are generally considered reliable; however, some recall errors may have been present.

### Statistical analysis

eTable 2 illustrates the distribution of baseline covariates that exhibited missing data, with the majority of these instances being below 4.70%. In the primary analysis, cases with missing data were excluded based on the assumption of missing completely at random. In general, our analytical approach comprised four steps: (1) comparing baseline characteristics; (2) assessing the risk of tooth loss progression for all-cause mortality; (3) conducting stratified analyses; and (4) performing sensitivity analyses.

Firstly, baseline characteristics were presented across the groups of tooth loss progression (i.e., stable, slow loss, moderate loss, and rapid loss). Continuous variables were described as median values with interquartile ranges (IQR), while categorical variables were reported as counts with corresponding percentages. The p-value for trend across the groups was computed using the Spearman test for continuous variables, while the p-value for trend in categorical variables was assessed using the Mantel-Haenszel test.

Secondly, Cox proportional-hazards model was used to estimate hazard ratios (HRs) and 95% confidence intervals (CIs) for the association between tooth loss progression and all-cause mortality. We did not find evidence suggesting potential violation of the proportional-hazards assumption for the exposure–outcome association, and two covariates with violation of the assumption were corrected through stratification, including current regular exercise and hypertension. In the Cox regression analysis, adjustments were performed in three stages: (1) an initial adjustment for sex and age; (2) the inclusion of additional covariates including education, marital status, residence, co-residence, current smoking, current drinking, current regular exercise, regular intake of foods, comorbidities, ADL disability, and denture; and (3) a further adjustment for baseline tooth count (the fully adjusted model). Meanwhile, an adjusted Kaplan-Meier survival analysis was conducted to illustrate prognosis trends across different groups. In addition, we fitted a cubic spline regression model with three knots to examine the relationship between annual tooth loss and mortality, and in the analysis, the annual tooth loss exceeding 12 teeth per year was coded as a value of 12 due to most instances of tooth loss being less than this threshold (eFigure 1).

Thirdly, the stratified analysis was conducted to assess the consistency of the association between tooth loss progression and mortality in various subgroups, with interactions examined through likelihood ratio testing. Finally, to assess the robustness of primary findings, we performed a series of sensitivity analyses, including: (1) to minimize potential reverse causation, deaths within the first year or two years of follow-up were excluded; (2) sensitivity analyses were performed for participants lost to follow-up, censoring at median and end of follow-up; (3) multiple imputation was used to handle missing data; (4) propensity score matching (PSM) was applied to ensure comparability between groups (“without tooth loss” vs. “with tooth loss”) regarding potential confounders; (5) a sensitivity analysis was conducted focusing on participants who had at least one tooth both at baseline and during re-examination, which aimed to minimize bias introduced by individuals without teeth at the time of re-examination; (6) E-values were used to evaluate the sensitivity to unmeasured or unknown confounding factors [23]; (7) exceptional cases were taken into account where older individuals might exhibit newly developed teeth at re-examination; and (8) incorporating participants with tooth increase, we treated the data on tooth increase as missing and performed multiple imputation to assess the association between tooth loss progression and mortality.

All analyses were performed with R version 4.1.0 including the “compareGroups”, “survival”, “tidyverse”, “rms”, “mice”, “forestplot”, “survminer”, and “stats” packages (http://www.R-project.org). All tests were two sided, and p values < 0.05 were considered statistically significant.

## Results

### Baseline characteristics

Table 1 presents baseline characteristics. The present study involved a total of 8073 older participants, with a median age of 83.0 years (IQR: 73.0–91.0). Among the participants, 3765 (46.6%) were men. The overall median values (IQR) for baseline tooth count, re-examined tooth count, and annual tooth loss were found to be 11.0 (5.0–23.0), 5.0 (1.0–12.0), and 1.4 (0.5–3.2), respectively. Participants exhibiting rapid tooth loss were more likely to be male, younger, better educated, and married. In addition, participants with rapid tooth loss tended to have a regular intake of fruit and beans. Other detailed information regarding baseline characteristics is also summarized in Table 1.

**Table 1:** Baseline characteristics

	All	Tooth loss progression				*p* for trend
		Stable	Slow loss	Moderate loss	Rapid loss	
No. of participants	8073	1272	3612	1649	1540	
Sex: male	3765 (46.6%)	576 (45.3%)	1605 (44.4%)	801 (48.6%)	783 (50.8%)	< 0.001
Age (years)	83.0 (73.0–91.0)	84.0 (75.0–92.0)	85.0 (74.0–93.0)	81.0 (71.0–90.0)	80.0 (71.0–88.0)	< 0.001
Education						0.002
No school	4768 (59.1%)	754 (59.3%)	2200 (60.9%)	963 (58.4%)	851 (55.3%)	
1 year or more	3305 (40.9%)	518 (40.7%)	1412 (39.1%)	686 (41.6%)	689 (44.7%)	
Marital status						< 0.001
Not in marriage	4752 (58.9%)	788 (61.9%)	2228 (61.7%)	940 (57.0%)	796 (51.7%)	
In marriage	3321 (41.1%)	484 (38.1%)	1384 (38.3%)	709 (43.0%)	744 (48.3%)	
Residence						0.712
Urban	3095 (38.3%)	492 (38.7%)	1377 (38.1%)	619 (37.5%)	607 (39.4%)	
Rural	4978 (61.7%)	780 (61.3%)	2235 (61.9%)	1030 (62.5%)	933 (60.6%)	
Co-residence						0.767
With family members	6789 (84.1%)	1065 (83.7%)	3050 (84.4%)	1372 (83.2%)	1302 (84.5%)	
Alone	1052 (13.0%)	164 (12.9%)	479 (13.3%)	223 (13.5%)	186 (12.1%)	
In a nursing home	232 (2.9%)	43 (3.4%)	83 (2.3%)	54 (3.3%)	52 (3.4%)	
Current smoking	1744 (21.6%)	289 (22.7%)	725 (20.1%)	384 (23.3%)	346 (22.5%)	0.263
Current drinking	1895 (23.5%)	323 (25.4%)	823 (22.8%)	389 (23.6%)	360 (23.4%)	0.491
Current regular exercise	2604 (32.3%)	427 (33.6%)	1134 (31.4%)	523 (31.7%)	520 (33.8%)	0.608
Regular intake of foods						
Fruit	2382 (29.5%)	335 (26.3%)	1044 (28.9%)	506 (30.7%)	497 (32.3%)	< 0.001
Vegetable	7007 (86.8%)	1111 (87.3%)	3109 (86.1%)	1440 (87.3%)	1347 (87.5%)	0.446
Meat	3364 (41.7%)	520 (40.9%)	1502 (41.6%)	694 (42.1%)	648 (42.1%)	0.493
Fish	1839 (22.8%)	264 (20.8%)	825 (22.8%)	377 (22.9%)	373 (24.2%)	0.051
Eggs	3567 (44.2%)	559 (43.9%)	1620 (44.9%)	711 (43.1%)	677 (44.0%)	0.620
Beans	3010 (37.3%)	433 (34.0%)	1333 (36.9%)	619 (37.5%)	625 (40.6%)	< 0.001
Comorbidities						
Hypertension	1320 (16.4%)	237 (18.6%)	588 (16.3%)	252 (15.3%)	243 (15.8%)	0.046
Diabetes	146 (1.8%)	13 (1.0%)	74 (2.0%)	22 (1.3%)	37 (2.4%)	0.078
Heart disease	613 (7.6%)	98 (7.7%)	284 (7.9%)	121 (7.3%)	110 (7.1%)	0.399
Cerebrovascular disease	325 (4.0%)	58 (4.6%)	132 (3.7%)	71 (4.3%)	64 (4.2%)	0.915
Respiratory disease	845 (10.5%)	149 (11.7%)	383 (10.6%)	174 (10.6%)	139 (9.0%)	0.027
ADL disability	1080 (13.4%)	188 (14.8%)	547 (15.1%)	181 (11.0%)	164 (10.6%)	< 0.001
Denture	1377 (17.1%)	212 (16.7%)	635 (17.6%)	310 (18.8%)	220 (14.3%)	0.115
Baseline tooth count	11.0 (5.0–23.0)	5.0 (2.0–14.0)	6.0 (3.0–15.0)	14.0 (9.0–24.0)	23.0 (17.0–28.0)	< 0.001
Re-examined tooth count	5.0 (1.0–12.0)	5.0 (2.0–14.0)	4.0 (0.0–12.0)	6.0 (0.0–15.0)	4.0 (0.0–10.0)	< 0.001
Annual tooth loss (teeth/year)	1.4 (0.5–3.2)	0.0 (0.0–0.0)	0.9 (0.5–1.4)	2.8 (2.3–3.3)	5.9 (4.8–7.6)	< 0.001

Values are median (IQR) or n (%)

*ADL* Activities of daily living, *IQR* Interquartile range

### Associations between tooth loss progression and all-cause mortality

**Fig. 2:**
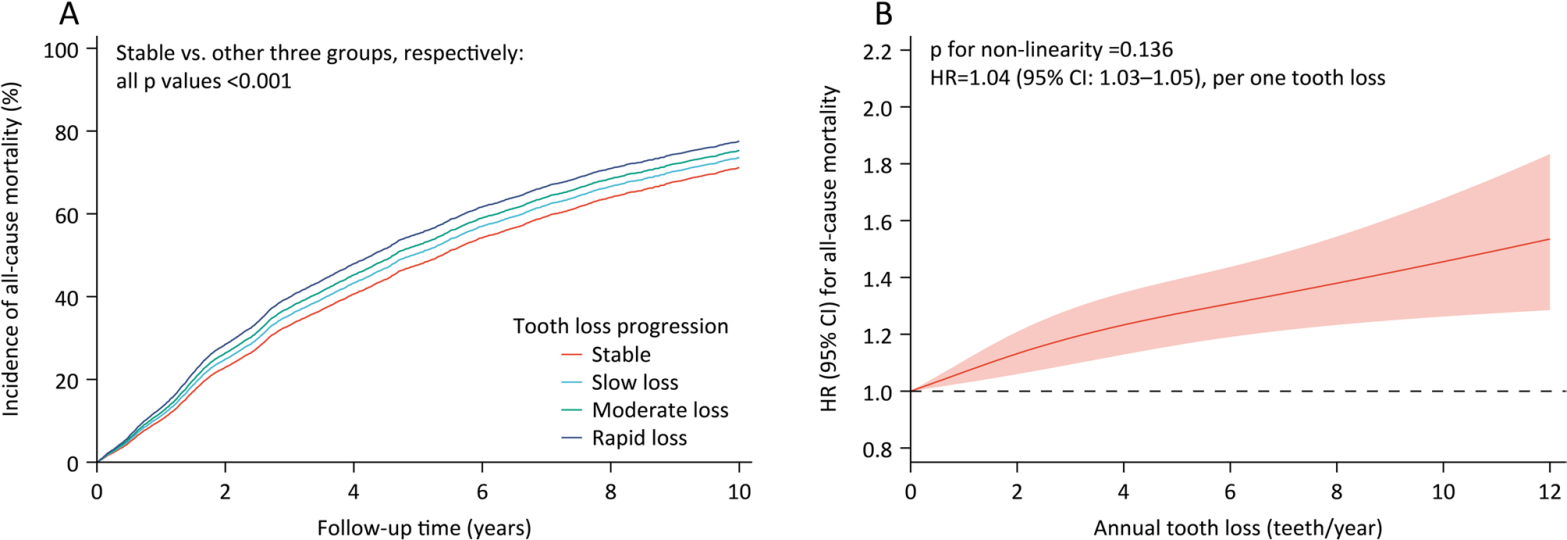
Associations between tooth loss progression and all-cause mortality. Note:(A) Cumulative mortality associated with varying levels of tooth loss, after adjusting for factors such as sex, age, education, marital status, residence, co-residence, current smoking, current drinking, current regular exercise, regular intake of foods (fruit, vegetable, meat, fish, eggs, and beans), comorbidities (hypertension, diabetes, heart disease, cerebrovascular disease, and respiratory disease), ADL disability, denture, and baseline tooth count. (B) To examine the association between annual tooth loss and all-cause mortality, we employed a Cox regression model incorporating a restricted cubic spline with three knots. This analysis was adjusted for the factors outlined in Figure A. The solid line represents the point estimates of HRs for mortality, while the shaded area indicates the corresponding 95% CIs. Abbreviations: ADL=activities of daily living, CI=confidence interval, HR=hazard ratio

During a median follow-up period of 3.5 years, 5176 (64.1%) deaths were recorded. After adjusting for baseline tooth count and other confounding variables, the risk of all-cause mortality significantly increased with a more rapid progression of tooth loss (Table 2; Fig. 2A). With the stable group as the reference, adjusted HRs for mortality were 1.11 (95% CI: 1.03–1.20) for the slow loss group, 1.20 (95% CI: 1.09–1.32) for the moderate loss group, and 1.33 (95% CI: 1.19–1.48) for the rapid loss group, respectively (Table 2). Furthermore, cubic spline analyses showed a positive linear relationship between annual tooth loss and mortality (adjusted HR = 1.04, 95% CI: 1.03–1.05, per one tooth loss; p for non-linearity = 0.136; Fig. 2B).

**Table 2:** Associations between tooth loss progression and all-cause mortality

	Tooth loss progression				*p* for trend^e^
	Stable	Slow loss	Moderate loss	Rapid loss	
No. of participants	1272	3612	1649	1540	
No. of deaths	843	2405	1017	911	
Follow-up (PYs)	5786.3	15672.2	7858.3	7499.6	
Mortality rate (95% CI)^a^	14.6 (13.7–15.5)	15.3 (14.8–15.9)	12.9 (12.2–13.7)	12.1 (11.4–12.9)	
Crude HR (95% CI), p	1.00 (ref)	1.05 (0.97–1.14), 0.194	0.89 (0.81–0.97), 0.012	0.84 (0.76–0.92), < 0.001	
Adjusted HR (95% CI), p					
model 1^b^	1.00 (ref)	1.09 (1.01–1.18), 0.033	1.10 (1.01–1.21), 0.038	1.13 (1.03–1.24), 0.013	
model 2^c^	1.00 (ref)	1.10 (1.01–1.19), 0.022	1.11 (1.01–1.22), 0.028	1.14 (1.03–1.25), 0.008	
model 3^d^	1.00 (ref)	1.11 (1.03–1.20), 0.009	1.20 (1.09–1.32), < 0.001	1.33 (1.19–1.48), < 0.001	< 0.001

*ADL* Activities of daily living, *CI* Confidence interval, *HR* Hazard ratio, *PYs* Person-years

^a^Per 100 PYs

^b^Model 1: adjusted for sex and age

^c^Model 2: further adjusted for other covariates, including education, marital status, residence, co-residence, current smoking, current drinking, current regular exercise, regular intake of foods (fruit, vegetable, meat, fish, eggs, and beans), comorbidities (hypertension, diabetes, heart disease, cerebrovascular disease, and respiratory disease), ADL disability, and denture

^d^Model 3: further adjusted for baseline tooth count

^e^The test was based on the median value for each group

In addition, a negative linear relationship was observed between baseline tooth count and all-cause mortality (p for non-linearity = 0.164; eFigure 2 A), and the adjusted HR was found to be 0.986 per two teeth increase (95% CI: 0.979–0.993). A similar relationship was noted between re-examined tooth count and mortality, with a p-value for non-linearity of 0.578; the adjusted HR was calculated at 0.972 (95% CI: 0.964–0.981; eFigure 2B).

### Stratified analysis

**Fig. 3:**
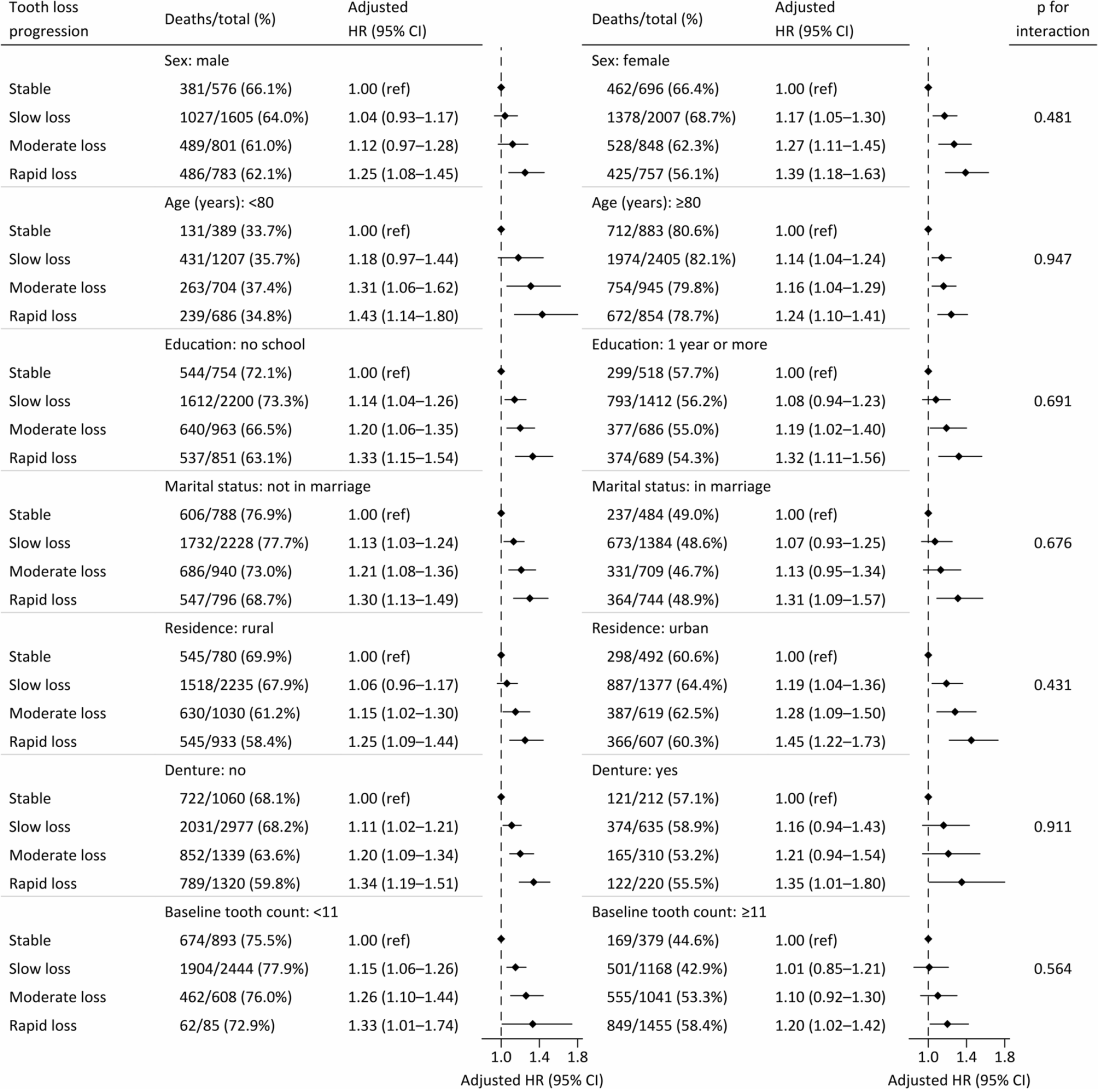
Stratified analyses by potential modifiers of the association between tooth loss progression and all-cause mortality. Note:Each stratification was adjusted for all factors, including sex, age, education, marital status, residence, co-residence, current smoking, current drinking, current regular exercise, regular intake of foods (fruit, vegetable, meat, fish, eggs, and beans), comorbidities (hypertension, diabetes, heart disease, cerebrovascular disease, and respiratory disease), ADL disability, denture, and baseline tooth count; however, the stratification factor itself was excluded from these adjustments. In the subgroup categorized by baseline tooth count, as determined by the median value, we further controlled for this variable itself.There were a substantial number of stratified analyses, and additional information was in the supplementary material (eFigure 3).Abbreviations: ADL=activities of daily living, CI=confidence interval, HR=hazard ratio

We performed stratified analyses to assess the association between tooth loss progression and all-cause mortality in various subgroups. None of the variables, including sex, age, education, marital status, residence, denture, number of baseline teeth, co-residence, lifestyle, comorbidities, and ADL disability, significantly modified the observed association in the primary analysis (Fig. 3, eFigure 3).

### Sensitivity analysis

In sensitivity analyses, all results were consistent with the primary findings, and the conclusions remained unchanged. The association between tooth loss progression and all-cause mortality persisted even after excluding participants who died within the first year or first two years of follow-up (eTable 3). Moreover, there were no substantial changes in the result when participants lost to follow-up were treated as censored at the median or end of follow-up (eTable 4). Similar results were also observed when missing data were handled through multiple imputation (eTable 5). Additionally, the association was unchanged when PSM was used (eTable 6). Furthermore, robustness of the findings was confirmed among participants who had at least one tooth both at baseline and at re-examination (eTable 7). The E-values indicated that the primary findings were unlikely to be nullified by an unmeasured or unknown confounding variable (eTables 8 and 9). Among participants with tooth increase between two successive waves, tooth increase tended to be associated with lower mortality risk (eTable 10). Finally, after incorporating participants with tooth increase and treating the data on tooth increase as missing, multiple imputation was then used to assess the association between tooth loss progression and all-cause mortality, yielding similar results with the primary results (eTable 11). Baseline characteristics of included participants and those with tooth increase are shown in eTable 12.

## Discussion

The present study, to the best of our knowledge, represents the first investigation into the association between tooth loss progression and mortality within a large prospective cohort of older adults. Our findings indicate that the risk of all-cause mortality significantly escalates with a more rapid progression of tooth loss, even after controlling for baseline tooth count and other confounding variables.

Previous studies of the association between tooth loss and mortality typically performed a single assessment of baseline tooth count [6, 8, 9, 11–13], and the present study extends those findings to demonstrate that tooth loss progression is also associated with all-cause mortality among older adults. The mechanism underlying the association of tooth loss with mortality remains uncertain, but several possible explanations could be considered. Firstly, higher inflammatory levels may mediate the association. A study suggested that poor oral health in older age, particularly tooth loss, was consistently associated with some inflammatory and cardiac biomarkers [24], and another two studies also found higher C-reactive protein levels to be possibly mediating the association between tooth loss and mortality [6]. Secondly, dietary aspects are another mechanism. The presence of healthy functioning teeth offers a better nutritional outcome, and tooth loss can compromise nutrition, further increasing the risk of mortality [25]. A recent study also showed nutritional status might contribute to the association between tooth loss and mortality in community-dwelling older Japanese individuals with fewer remaining teeth [26]. Thirdly, tooth loss can cause psychological distress and social isolation, thereby contributing to poorer mental health, and ultimately increase mortality [27]. Fourthly, tooth loss has been found to be associated with obesity [28], frailty [29], and physical and cognitive decline [30], all of which have been associated with an increased risk of mortality. However, despite these explanations suggesting an association between tooth loss and other known mortality risk factors, the exact mechanisms remain unclear and warrant further investigation. Additionally, it is important to acknowledge that the present findings are based on an observational study which may introduce potential biases. Nonetheless, the strong statistical significance and the consistent results obtained from sensitivity analyses reduce the likelihood of this possibility.

On the other hand, some researchers have proposed that the association might not be causal [6, 31], and it might be more plausible that the association between tooth loss and mortality reflects the fact that oral and general health share many mortality risk factors [2, 32, 33]. There’s no denying that tooth loss should be considered in the context of socioeconomic demographics, because people who are poorer, less educated and engage in unhealthy habits are more likely to have fewer teeth and be less healthy than those who do not experience the same limitations; meanwhile, severe tooth loss is more common in people with each chronic condition than in people who do not have the condition [32]. All of these general health issues, along with tooth loss, may raise mortality risk.

In our view, even if current evidence might be impossible to clearly identify the causal association, at least it is significantly associative. Tooth loss may represent an integrative measure of multiple socio-economic and physiological processes, explaining its associations with mortality. Therefore, tooth loss progression may serve as an indicator of deterioration in these multiple processes, and monitoring the progression could be valuable in older adults. Additionally, several modifiable factors such as lack of dental check-ups, infrequent brushing, and smoking have been found to influence the progression of tooth loss [18, 19]. Moreover, some of these modifiable factors like regular tooth brushing and dental visits have shown an inverse association with mortality among elderly individuals with tooth loss [34]. Therefore, the integration of these modifiable factors into public programs aimed at mitigating tooth loss may have the potential to further decrease mortality risk. Achieving this goal requires a harmonization of priorities across oral health, public health, and healthcare systems, as well as in related education, training, research, and health policy initiatives. This endeavor demands sustained and concerted political support alongside the active involvement of all stakeholders, including patients and communities. Furthermore, is it necessary to replace missing teeth? While research on this topic remains limited, some studies suggest that the use of dentures may be associated with a lower risk of mortality [9, 35]. In our study, the point estimate indicated a potential benefit of dentures in reducing mortality risk within the fully adjusted model (HR = 0.96, 95% CI: 0.88–1.04; data not shown), although this finding did not achieve statistical significance. The reasons for discrepancies with previous studies remain unclear but may be attributed to variations in confounding factors and population characteristics. Nevertheless, current evidence may support the possible role of dentures in mitigating mortality risk.

Strengths of our study include the large sample size, its prospective cohort study design, and a long follow-up time, as well as using longitudinal assessment of tooth count to examine the association of tooth loss progression with mortality. This study also has some limitations. Firstly, caution is needed when generalizing our findings to other age groups, regions or ethnicities as our study population primarily consisted of older adults in China. Secondly, while self-reported tooth count demonstrates a strong correlation with actual oral examinations [36], it may still introduce bias, particularly if participants either underreport or overreport their tooth count. This limitation is inherent in the context of real-world epidemiological studies. Nevertheless, the association between tooth count assessed at a single point and mortality is consistent with findings from numerous previous studies, suggesting that the data should be considered relatively reliable. Thirdly, the lack of other dimensions of oral health, such as periodontal status, limited further investigations. Fourthly, over 4000 participants were excluded due to tooth increase, and this might reflect various conditions, including exceptional cases of natural tooth increase, dental interventions—including implants and dentures—self-reported inaccuracies, or other phenomena; however, a specific assessment cannot be conducted based on the available data. The main concern is that excluding these participants may have introduced selection bias, potentially leading to an overestimation or underestimation of HRs for mortality associated with tooth loss. Additionally, this large-scale exclusion may significantly affect the generalizability of our findings. To address the issue, we defined the annual tooth increase as missing for participants with such increases and combined them with the 8073 study participants to create a comprehensive dataset. After multiple imputation, the results were consistent with the primary findings. Finally, the possible confounding from unmeasured factors cannot completely be ruled out despite the fact that we had adjusted extensively for baseline characteristics. While, the E-value analysis could suggest the robustness of the findings in some degree. Despite these limitations, our results still clearly indicate that tooth loss progression was significantly associated with mortality.

## Conclusion

The risk of all-cause mortality significantly increased with a more rapid progression of tooth loss, even after adjusting for baseline tooth count and other confounders. The present findings emphasize the importance and necessity of closely monitoring the progression of tooth loss among older adults. It is reasonable to suggest that healthcare professionals and the general public should be aware of the potential adverse prognosis associated with a rapid progression of tooth loss.

## Supplementary Information

Supplementary Material 1.

## Data Availability

Researchers can download the datasets free of charge from the following websites: (1) https://opendata.pku.edu.cn; Peking University Open Access Research Database; (2) https://www.icpsr.umich.edu/icpsrweb/NACDA/series/487; National Archive of Computerized Data on Aging (NACDA) sponsored by U.S. National Institute of Aging (NIA/NIH), Inter-university Consortium for Political and Social Research (ICPSR) at University of Michigan.

## References

[1] Bernabe E, Marcenes W, Hernandez CR, Bailey J, Abreu LG, Alipour V, et al. Global, regional, and national levels and trends in burden of oral conditions from 1990 to 2017: a systematic analysis for the global burden of disease 2017 study. J Dent Res. 2020;99(4):362–73. 32122215 10.1177/0022034520908533 PMC7088322

[2] Jain N, Dutt U, Radenkov I, Jain S. WHO’s global oral health status report 2022: actions, discussion and implementation. Oral Dis. 2023. 10.1111/odi.14516. 36680388 10.1111/odi.14516

[3] Haugejorden O, Klock KS, Trovik TA. Incidence and predictors of self-reported tooth loss in a representative sample of Norwegian adults. Community Dent Oral Epidemiol. 2003;31(4):261–8. 12846848 10.1034/j.1600-0528.2003.00004.x

[4] Murray Thomson W. Epidemiology of oral health conditions in older people. Gerodontology. 2014;31(Suppl 1):9–16. 24446974 10.1111/ger.12085

[5] Dye B, Thornton-Evans G, Li X, Iafolla T. Dental caries and tooth loss in adults in the united states, 2011–2012. NCHS Data Brief 2015(197):197. 25973996

[6] Koka S, Gupta A. Association between missing tooth count and mortality: a systematic review. J Prosthodont Res. 2018;62(2):134–51. 28869174 10.1016/j.jpor.2017.08.003

[7] Peres MA, Macpherson LMD, Weyant RJ, Daly B, Venturelli R, Mathur MR, et al. Oral diseases: a global public health challenge. Lancet. 2019;394(10194):249–60. 31327369 10.1016/S0140-6736(19)31146-8

[8] Yuan J-Q, Lv Y-B, Kraus VB, Gao X, Yin Z-X, Chen H-S, et al. Number of natural teeth, denture use and mortality in Chinese elderly: a population-based prospective cohort study. BMC Oral Health. 2020;20(1):100. 32276615 10.1186/s12903-020-01084-9 PMC7147045

[9] Dai M, Song Q, Lin T, Huang X, Xie Y, Wang X, et al. Tooth loss, denture use, and all-cause and cause-specific mortality in older adults: a community cohort study. Front Public Health. 2023. 10.3389/fpubh.2023.1194054. 37342280 10.3389/fpubh.2023.1194054 PMC10277727

[10] Kassebaum NJ, Bernabe E, Dahiya M, Bhandari B, Murray CJ, Marcenes W. Global burden of severe tooth loss: a systematic review and meta-analysis. J Dent Res. 2014;93(7 Suppl):S20-8. 10.1177/0022034514537828 PMC4293725 24947899

[11] Goto Y, Wada K, Uji T, Koda S, Mizuta F, Yamakawa M, et al. Number of teeth and all-cause and cancer mortality in a Japanese community: the Takayama study. J Epidemiol. 2020;30(5):213–8. 31006716 10.2188/jea.JE20180243 PMC7153964

[12] Abnet CC, Qiao Y-L, Dawsey SM, Dong Z-W, Taylor PR, Mark SD. Tooth loss is associated with increased risk of total death and death from upper gastrointestinal cancer, heart disease, and stroke in a Chinese population-based cohort. Int J Epidemiol. 2005;34(2):467–74. 15659476 10.1093/ije/dyh375

[13] Manabe K, Tanji F, Tomata Y, Zhang S, Tsuji I. Preventive effect of oral self-care on pneumonia death among the elderly with tooth loss: the Ohsaki cohort 2006 study. Tohoku J Exp Med. 2019;247(4):251–7. 30996210 10.1620/tjem.247.251

[14] Copeland LB, Krall EA, Brown LJ, Garcia RI, Streckfus CF. Predictors of tooth loss in two US adult populations. J Public Health Dent. 2004;64(1):31–7. 15078059 10.1111/j.1752-7325.2004.tb02723.x

[15] Silva-Junior MF, Batista MJ, de Sousa MLR. Incidence of tooth loss in adults: A 4-Year Population-Based prospective cohort study. Int J Dent 2017, 2017:1–7. 10.1155/2017/6074703 PMC5529659 28785282

[16] Needleman I, Garcia R, Gkranias N, Kirkwood KL, Kocher T, Iorio AD, et al. Mean annual attachment, bone level, and tooth loss: a systematic review. J Periodontol. 2018;89(Suppl 1):S120-39. 29926956 10.1002/JPER.17-0062

[17] Wang H, Chen H. Aging in China: challenges and opportunities. China CDC Wkly. 2022;4(27):601–2. 35919296 10.46234/ccdcw2022.130 PMC9339359

[18] Susin C, Oppermann RV, Haugejorden O, Albandar JM. Tooth loss and associated risk indicators in an adult urban population from South Brazil. Acta Odontol Scand. 2009;63(2):85–93. 10.1080/00016350510019694 16134547

[19] Ishikawa S, Konta T, Susa S, Kitabatake K, Ishizawa K, Togashi H, et al. Risk factors for tooth loss in community-dwelling Japanese aged 40 years and older: the Yamagata (Takahata) study. Clin Oral Invest. 2018;23(4):1753–60. 10.1007/s00784-018-2604-x 30167794

[20] Zeng Y. Towards deeper research and better policy for healthy aging --Using the unique data of Chinese longitudinal healthy longevity survey. China Econ J. 2012;5(2–3):131–49. 10.1080/17538963.2013.764677 PMC3893304 24443653

[21] Zeng Y, Feng Q, Hesketh T, Christensen K, Vaupel JW. Survival, disabilities in activities of daily living, and physical and cognitive functioning among the oldest-old in China: a cohort study. Lancet. 2017;389(10079):1619–29. 28285816 10.1016/S0140-6736(17)30548-2 PMC5406246

[22] Wang Z, Zheng Y, Ruan H, Li L, Duan L, He S. Association between social activity frequency and overall survival in older people: results from the Chinese longitudinal healthy longevity survey (CLHLS). J Epidemiol Community Health. 2023;77(5):277–84. 36878718 10.1136/jech-2022-219791

[23] VanderWeele TJ, Ding P. Sensitivity analysis in observational research: introducing the E-value. Ann Intern Med. 2017;167(4):268–74. 28693043 10.7326/M16-2607

[24] Kotronia E, Wannamethee SG, Papacosta AO, Whincup PH, Lennon LT, Visser M, Kapila YL, Weyant RJ, Ramsay SE, Melzer D. Poor oral health and inflammatory, hemostatic, and cardiac biomarkers in older age: results from two studies in the UK and USA. Journals Gerontology: Ser A. 2021;76(2):346–51. 10.1093/gerona/glaa096 PMC7812424 32306041

[25] Ikebe K, Morii K, Matsuda K, Nokubi T. Discrepancy between satisfaction with mastication, food acceptability, and masticatory performance in older adults. Int J Prosthodont. 2007;20(2):161–7. 17455437

[26] Hiratsuka T, Komiyama T, Ohi T, Tanji F, Tomata Y, Tsuji I, et al. Contribution of systemic inflammation and nutritional status to the relationship between tooth loss and mortality in a community-dwelling older Japanese population: a mediation analysis of data from the Tsurugaya project. Clin Oral Invest. 2019;24(6):2071–7. 10.1007/s00784-019-03072-y 31485781

[27] Yamamoto T, Aida J, Kondo K, Fuchida S, Tani Y, Saito M, et al. Oral health and incident depressive symptoms: JAGES project longitudinal study in older Japanese. J Am Geriatr Soc. 2017;65(5):1079–84. 28165614 10.1111/jgs.14777

[28] Nascimento GG, Leite FR, Conceicao DA, Ferrua CP, Singh A, Demarco FF. Is there a relationship between obesity and tooth loss and edentulism? A systematic review and meta-analysis. Obes Rev. 2016;17(7):587–98. 27125768 10.1111/obr.12418

[29] Zhang X-M, Jiao J, Cao J, Wu X. The association between the number of teeth and frailty among older nursing home residents: a cross-sectional study of the CLHLS survey. BMC Geriatr. 2022. 10.1186/s12877-022-03688-y. 36585614 10.1186/s12877-022-03688-y PMC9805096

[30] Tsakos G, Watt RG, Rouxel PL, de Oliveira C, Demakakos P. Tooth loss associated with physical and cognitive decline in older adults. J Am Geriatr Soc. 2015;63(1):91–9. 25523131 10.1111/jgs.13190

[31] Araujo CF, Schuch HS, Cademartori MG, Bielemann RM, Bertoldi AD, Tomasi E, Gonzalez Maria C, Demarco FF. Functional dentition and edentulism associated with mortality: A cohort study of older adults in Southern Brazil. Commun Dent Oral Epidemiol. 2023;51(6):1209–15. 10.1111/cdoe.12862 37186382

[32] Parker ML, Thornton-Evans G, Wei L, Griffin SO. Prevalence of and changes in tooth loss among adults aged >/=50 years with selected chronic conditions - United states, 1999–2004 and 2011–2016. MMWR Morb Mortal Wkly Rep. 2020;69(21):641–6. 32463807 10.15585/mmwr.mm6921a1 PMC7269607

[33] Qi L, Qian Y, Zhu F, Cao N, Lu H, Zhang L. Association between periodontal disease and tooth loss and mortality in an elderly Chinese population. Aging Clin Exp Res. 2020;32(11):2375–82. 32020486 10.1007/s40520-019-01446-6

[34] Hayasaka K, Tomata Y, Aida J, Watanabe T, Kakizaki M, Tsuji I. Tooth loss and mortality in elderly Japanese adults: effect of oral care. J Am Geriatr Soc. 2013;61(5):815–20. 23590405 10.1111/jgs.12225

[35] Sabbah W, Slade GD, Sanders AE, Bernabé E. Denture wearing and mortality risk in edentulous American adults: a propensity score analysis. J Dent. 2020. 10.1016/j.jdent.2020.103360. 32404256 10.1016/j.jdent.2020.103360

[36] Buhlin K, Gustafsson A, Andersson K, Hakansson J, Klinge B. Validity and limitations of self-reported periodontal health. Community Dent Oral Epidemiol. 2002;30(6):431–7. 12453114 10.1034/j.1600-0528.2002.00014.x

