# Supplementary material for: Tooth loss progression and mortality among older adults: results from the Chinese longitudinal healthy longevity survey (CLHLS)
Source: BMC Geriatr. 2025 Oct 10;25:769. doi: 10.1186/s12877-025-06419-1 (PMC12512688; doi:10.1186/s12877-025-06419-1)
Supplement: Supplementary file 1 — Supplementary Material 1. [file 12877_2025_6419_MOESM1_ESM.pdf]

Tooth loss progression and mortality among older adults: results from the Chinese Longitudinal Healthy Longevity Survey (CLHLS)

Linjia Duan[1, 4], Liu Yang[2], Haiyan Ruan[1, 3], Halmira Alimjan[2], Liming Zhao[5], Ziqiong Wang[1], Lu Liu[1], Ningying Song[6*], Sen He[1, 2*]

1. Department of Cardiology, West China Hospital, Sichuan University, Chengdu, China.
2. Department of Cardiology, Karamay Hospital of Integrated Chinese and Western Medicine, Karamay, China.
3. Department of Cardiology, Hospital of Traditional Chinese Medicine, Shuangliu District, Chengdu, China.
4. West China School of Nursing, Sichuan University, Chengdu, China
5. Department of Cardiology, Hospital of Chengdu Office of People's Government of Tibetan Autonomous Region, Chengdu, China
6. Department of Otolaryngology-Head & Neck Surgery, West China Hospital, Sichuan University, Chengdu, China.

[*]Corresponding author:

Sen He: Department of Cardiology, West China Hospital, Sichuan University, Chengdu, China; E-mail: hesen_sky@scu.edu.cn.

Ningying Song: Department of Otolaryngology-Head & Neck Surgery, West China Hospital, Sichuan University, Chengdu, China; E-mail: songningying2@163.com.

This study was supported by the Sichuan Science and Technology Program, China (Grant No. 2022YFS0186), the National Natural Science Foundation of China (Grant No. 81600299), the Science and Technology Major Project of Tibetan Autonomous Region of China (Grant No. XZ202201ZD0001G01), and the Key Research and Development Projects of Chengdu Science and Technology Bureau (Grant No. 2022-YF05–01335-SN).

List of supplementary materials

eTable 1. Definitions of baseline covariates in the present study 3
eTable 2. Distributions of baseline covariates with missing data 6
eTable 3. Associations between tooth loss progression and all-cause mortality after excluding deaths within the first year or the first two years 7
eTable 4. Associations between tooth loss progression and all-cause mortality, in considering the losses censored at varying time of follow-up (n=9588) 7
eTable 5. Associations between tooth loss progression and all-cause mortality after multiple imputation (n=8797)[a] 7
eTable 6. Associations between tooth loss progression and all-cause mortality in the PSM sample (n=3739)[a] 8
eTable 7. Associations between tooth loss progression and all-cause mortality in participants who had at least one tooth at baseline and at re-examination (n=6166) 8
eTable 8. E-values for the fully adjusted HRs (model 3) in Table 2 8
eTable 9. Multivariate Cox regression analysis of all-cause mortality 9
eTable 10. Associations between tooth increase progression[a] and all-cause mortality in participants who had increased tooth count between two successive waves (n=9507) 10
eTable 11. Incorporating participants with tooth increase, treating the data on tooth increase as missing, and using multiple imputation to assess the association between tooth loss progression and all-cause mortality[a] (n=12440) 10
eTable 12. Baseline characteristics of included participants and those with tooth increase 11
eFigure 1. Distributions of annual tooth loss 12
eFigure 2. Associations between baseline tooth count and re-examined tooth count and all-cause mortality 12
eFigure 3. Stratified analyses by potential modifiers of the association between tooth loss progression and all-cause mortality (Figure 3 continued) 13

eTable 1. Definitions of baseline covariates in the present study

	Questions<br>in the CLHLS questionnaire	Options for the question	Scales of reclassification<br>in the present study
Sex		· male<br>· female	· Male: male<br>· Female: female
Age			· Continuous (years)
Education	How many years did you attend school?	· years of school<br>· don't know<br>· missing	· No school: years of school =0<br>· 1 year or more: years of school ≥1<br>· missing: don't know, missing
Marital status	Current marital status?	· currently married and living with spouse<br>· separated<br>· divorced<br>· widowed<br>· never married<br>· don't know<br>· missing	· In marriage:<br>currently married and living with spouse, separated<br>· Not in marriage:<br>divorced, widowed, never married<br>· missing: don't know, missing
Residence	Current residence area of interviewee?	Wave 1998<br>· urban (city and town)<br>· rural<br><br>Waves 2000, 2002, 2005, 2008, 2011<br>· city<br>· town<br>· rural	· Urban: city, town<br>· Rural: rural
Co-residence	Co-residence?	· with family member(s)<br>· alone<br>· in a nursing home<br>· missing	· With family members: with household member(s)<br>· Alone: alone<br>· In a nursing home: in a nursing home<br>· missing: missing
Current smoking	Do you smoke at present?	· yes<br>· no<br>· missing	· Current smoking: yes<br>· No smoking at present: no<br>· missing
Current drinking	Do you drink at present?	· yes<br>· no<br>· don't know<br>· missing	· Current drinking: yes<br>· No drinking at present: no<br>· missing: don't know, missing
Current regular exercise	Do you do exercises regularly at present?	· yes<br>· no<br>· don't know<br>· missing	· Current regular exercise: yes<br>· No regular exercise at present: no<br>· missing: don't know, missing
Regular intake of fruit, and vegetable, respectively	Do you eat these foods, respectively?	· almost everyday<br>· except winter/quite often	· Regular intake:<br>almost everyday, except winter/quite often

	Questions in the CLHLS questionnaire	Options for the question	Scales of reclassification in the present study
		· occasionally<br>· rarely or never<br>· don't know<br>· missing	· No regular intake:<br>occasionally, rarely or never<br>· missing: don't know, missing
Regular intake of meat, fish, eggs, and beans, respectively	Do you eat these foods, respectively?	Waves 1998, 2000, 2002, 2005<br>· almost everyday<br>· occasionally<br>· rarely or never<br>· missing<br><br>Waves 2008, 2011<br>· almost everyday<br>· not everyday, but at least once per week<br>· not every week, but at least once per month<br>· not every month, but occasionally<br>· rarely or never<br>· don't know<br>· missing	· Regular intake:<br>almost everyday; not everyday, but at least once per week<br>· No regular intake:<br>not every week, but at least once per month; not every month, but occasionally; occasionally; rarely or never.<br>· missing: don't know, missing
Hypertension, diabetes, heart disease, cerebrovascular disease, and respiratory disease, respectively	Are you suffering from these diseases, respectively?	· yes<br>· no<br>· don't know<br>· missing	· Yes: yes<br>· No: no<br>· missing: don't know, missing
ADL disability	Bathing:<br>Without assistance?	· without assistance<br>· one part assistance<br>· more than one part assistance<br>· don't know<br>· missing	· In the CLHLS survey, six items of daily self-care ability were collected from each participant based on the Katz index: dressing, bathing, transferring, toileting, continence, and eating. Each item included three answers: complete independence, partially dependence, and complete dependence.<br>ADL disability was defined as present if participants needed any assistance in performing at least one of the six self-care activities.<br>· missing: don't know, missing
	Dressing:<br>Get clothes and get completely dressed without assistance?	· without assistance<br>· need assistance for trying shoes<br>· assistance in getting clothes and getting dressed<br>· missing	
	Toileting:<br>Go to the toilet, cleans self, and arranges clothes without assistance (may use object for support such as cane, walker, or wheelchair)?	· without assistance<br>· assistance in cleaning or arranging clothes<br>· don't use toilet<br>· missing	
	Transferring:<br>Get in and out of bed as well as in and out of a chair without assistance (may use object for support such as cane or walker)?	· without assistance<br>· with assistance<br>· bedridden<br>· missing	

	Questions<br>in the CLHLS questionnaire	Options for the question	Scales of reclassification<br>in the present study
	Continence:<br>Has complete control of urination and bowel movement without assistance?	· without assistance<br>· occasional accidents<br>· incontinent<br>· missing	
	Feeding:<br>Feed self without assistance?	· without assistance<br>· with some help<br>· need feeding<br>· missing	
Denture	Do you have denture?	· yes<br>· no<br>· don't know<br>· missing	· Yes: yes<br>· No: no<br>· missing: don't know, missing

Note:
More detailed information about these covariates can be found on: https://agingcenter.duke.edu/CLHLS.
Abbreviations: ADL=activities of daily living, CLHLS=Chinese Longitudinal Healthy Longevity Survey.

eTable 2. Distributions of baseline covariates with missing data

	Number of missing data	Percentage of missing data (%)
Sex	0	0.00
Age	0	0.00
Education	42	0.40
Marital status	4	0.04
Residence	0	0.00
Co-residence	10	0.10
Current smoking	3	0.03
Current drinking	3	0.03
Current regular exercise	12	0.12
Regular intake of foods		
Fruit	6	0.06
Vegetable	10	0.10
Meat	18	0.17
Fish	30	0.29
Eggs	23	0.22
Beans	16	0.15
Comorbidities		
Hypertension	459	4.40
Diabetes	490	4.70
Heart disease	466	4.47
Cerebrovascular disease	460	4.41
Respiratory disease	406	3.89
ADL disability	32	0.31
Denture	19	0.18

Note:
In the primary analysis, cases with missing data were excluded under the assumption of missing completely at random. However, we also performed multiple imputation as a sensitivity analysis to assess the impact of missing data on the association between exposures and outcomes.
Abbreviations: ADL=activities of daily living.

eTable 3. Associations between tooth loss progression and all-cause mortality after excluding deaths within the first year or the first two years

	Excluding deaths within the first year (n=7150)		Excluding deaths within the first two years (n=6018)	
	Adjusted HR (95% CI)[a]	p value	Adjusted HR (95% CI)[a]	p value
Stable	1.00 (ref)		1.00 (ref)	
Slow loss	1.09 (1.00–1.19)	0.042	1.07 (0.97–1.19)	0.175
Moderate loss	1.19 (1.08–1.33)	0.001	1.20 (1.07–1.35)	0.003
Rapid loss	1.28 (1.13–1.44)	<0.001	1.22 (1.06–1.39)	0.005

Note:

[a] With adjustment for sex, age, education, marital status, residence, co-residence, current smoking, current drinking, current regular exercise, regular intake of foods (fruit, vegetable, meat, fish, eggs, and beans), comorbidities (hypertension, diabetes, heart disease, cerebrovascular disease, and respiratory disease), ADL disability, denture, and baseline tooth count.

Abbreviations: ADL=activities of daily living, CI=confidence interval, HR=hazard ratio.

eTable 4. Associations between tooth loss progression and all-cause mortality, in considering the losses censored at varying time of follow-up (n=9588)

	Considering the losses censored at the median of follow-up (3.5 years)		Considering the losses censored at the end of follow-up (10.0 years)	
	Adjusted HR (95% CI)[a]	p value	Adjusted HR (95% CI)[a]	p value
Stable	1.00 (ref)		1.00 (ref)	
Slow loss	1.09 (1.01–1.18)	0.035	1.03 (0.96–1.12)	0.411
Moderate loss	1.15 (1.05–1.27)	0.004	1.07 (0.97–1.18)	0.157
Rapid loss	1.25 (1.12–1.40)	<0.001	1.13 (1.01–1.26)	0.027

Note:

[a] With adjustment for sex, age, education, marital status, residence, co-residence, current smoking, current drinking, current regular exercise, regular intake of foods (fruit, vegetable, meat, fish, eggs, and beans), comorbidities (hypertension, diabetes, heart disease, cerebrovascular disease, and respiratory disease), ADL disability, denture, and baseline tooth count.

Abbreviations: ADL=activities of daily living, CI=confidence interval, HR=hazard ratio.

eTable 5. Associations between tooth loss progression and all-cause mortality after multiple imputation (n=8797)[a]

	Adjusted HR (95% CI)[b]	p value
Stable	1.00 (ref)	
Slow loss	1.09 (1.01–1.17)	0.029
Moderate loss	1.18 (1.08–1.30)	<0.001
Rapid loss	1.33 (1.20–1.48)	<0.001

Note:

[a] Multiple imputation was performed by chained equations to create five datasets, of which the resultant model estimates for each were combined using Rubin`s rules. The present sample size (n=8797) was smaller than the calculated value in the flow chart ($8073_{\text{final sample}}$ plus $845_{\text{missing information on other covariates}}$ =8918), which was caused by the partial overlap between the lost participants and the participants with missing information on other covariates.

[b] With adjustment for sex, age, education, marital status, residence, co-residence, current smoking, current drinking, current regular exercise, regular intake of foods (fruit, vegetable, meat, fish, eggs, and beans), comorbidities (hypertension, diabetes, heart disease, cerebrovascular disease, and respiratory disease), ADL disability, denture, and baseline tooth count.

Abbreviations: ADL=activities of daily living, CI=confidence interval, HR=hazard ratio.

eTable 6. Associations between tooth loss progression and all-cause mortality in the PSM sample (n=3739)[a]

	Adjusted HR (95% CI)[b]	p value
Stable	1.00 (ref)	
Slow loss	1.13 (1.03–1.23)	0.009
Moderate loss	1.23 (1.07–1.40)	0.003
Rapid loss	1.32 (1.11–1.58)	0.002

Note:

[a] The two groups, namely "without tooth loss between two waves" vs. "with tooth loss between two waves", were matched by PSM, which was performed using the nearest neighbor matching algorithm, with a fixed caliper of 0.1 (1:2 matching, without replacement). After implementing PSM, the distributions of propensity scores between the two groups exhibited significant overlap (data not shown). Additionally, the ASD for each covariate between the groups was found to be less than 0.100 (data not shown), which is a widely accepted criterion for indicating balance.

[b] To eliminate the risk of insufficient covariate balance, we further adjusted for baseline covariates, with adjustment with sex, age, education, marital status, residence, co-residence, current smoking, current drinking, current regular exercise, regular intake of foods (fruit, vegetable, meat, fish, eggs, and beans), comorbidities (hypertension, diabetes, heart disease, cerebrovascular disease, and respiratory disease), ADL disability, denture, and baseline tooth count.

Abbreviations: ADL=activities of daily living, ASD=Absolute standardized mean differences, CI=confidence interval, HR=hazard ratio, PSM=propensity score matching.

eTable 7. Associations between tooth loss progression and all-cause mortality in participants who had at least one tooth at baseline and at re-examination (n=6166)

	Adjusted HR (95% CI)[a]	p value
Stable	1.00 (ref)	
Slow loss	1.11 (1.02–1.21)	0.017
Moderate loss	1.21 (1.09–1.35)	0.001
Rapid loss	1.28 (1.13–1.46)	<0.001

Note:

[a] With adjustment for sex, age, education, marital status, residence, co-residence, current smoking, current drinking, current regular exercise, regular intake of foods (fruit, vegetable, meat, fish, eggs, and beans), comorbidities (hypertension, diabetes, heart disease, cerebrovascular disease, and respiratory disease), ADL disability, denture, and baseline tooth count.

Abbreviations: ADL=activities of daily living, CI=confidence interval, HR=hazard ratio.

eTable 8. E-values for the fully adjusted HRs (model 3) in Table 2

	Adjusted HR (95% CI)[a], p	E-value
Stable	1.00 (ref)	
Slow loss	1.11 (1.03–1.20), 0.009	1.46
Moderate loss	1.20 (1.09–1.32), <0.001	1.69
Rapid loss	1.33 (1.19–1.48), <0.001	1.99

Note:

[a] With adjustment for sex, age, education, marital status, residence, co-residence, current smoking, current drinking, current regular exercise, regular intake of foods (fruit, vegetable, meat, fish, eggs, and beans), comorbidities (hypertension, diabetes, heart disease, cerebrovascular disease, and respiratory disease), ADL disability, denture, and baseline tooth count.

Abbreviations: ADL=activities of daily living, CI=confidence interval, HR=hazard ratio.

eTable 9. Multivariate Cox regression analysis of all-cause mortality

	Change	Adjusted HR (95% CI)[a]	p value
Sex	female vs. male	0.71 (0.66–0.76)	<0.001
Age (years)	per 1-year increment	1.07 (1.07–1.08)	<0.001
Education			
No school		1.00 (ref)	
1 year or more		0.95 (0.88–1.02)	0.129
Marital status			
Not in marriage		1.00 (ref)	
In marriage		0.86 (0.80–0.92)	<0.001
Residence			
Urban		1.00 (ref)	
Rural		0.99 (0.93–1.05)	0.705
Co-residence			
With family members		1.00 (ref)	
Alone		0.97 (0.90–1.06)	0.549
In a nursing home		1.23 (1.06–1.43)	0.005
Current smoking	yes vs. no	1.05 (0.98–1.14)	0.170
Current drinking	yes vs. no	1.01 (0.94–1.08)	0.774
Current regular exercise	yes vs. no	NA[b]	
Regular intake of foods			
Fruit	yes vs. no	0.94 (0.88–1.01)	0.100
Vegetable	yes vs. no	1.00 (0.92–1.08)	0.990
Meat	yes vs. no	0.97 (0.91–1.04)	0.426
Fish	yes vs. no	0.99 (0.91–1.07)	0.752
Eggs	yes vs. no	0.90 (0.85–0.96)	0.002
Beans	yes vs. no	1.01 (0.95–1.07)	0.814
Comorbidities			
Hypertension	yes vs. no	NA[b]	
Diabetes	yes vs. no	1.04 (0.82–1.33)	0.738
Heart disease	yes vs. no	0.99 (0.88–1.11)	0.885
Cerebrovascular disease	yes vs. no	1.27 (1.09–1.47)	0.002
Respiratory disease	yes vs. no	1.19 (1.09–1.29)	<0.001
ADL disability	yes vs. no	1.40 (1.29–1.51)	<0.001
Denture	yes vs. no	0.96 (0.88–1.04)	0.266
Baseline tooth count	yes vs. no	0.99 (0.98–0.99)	<0.001

Note:

[a] Each HR was adjusted for all factors (sex, age, education, marital status, residence, co-residence, current smoking, current drinking, current regular exercise, regular intake of foods [fruit, vegetable, meat, fish, eggs, and beans], comorbidities [hypertension, diabetes, heart disease, cerebrovascular disease, and respiratory disease], ADL disability, denture, baseline tooth count, and tooth loss progression) except the factor itself.

[b] Violation of the proportional-hazards assumption was found for these variables, with correction through stratification; therefore, HRs were not available for these variables.

Abbreviations: ADL=activities of daily living, CI=confidence interval, HR=hazard ratio.

eTable 10. Associations between tooth increase progression[a] and all-cause mortality in participants who had increased tooth count between two successive waves (n=9507)

	Adjusted HR (95% CI)[b]	p value
Stable	1.00 (ref)	
Slow increase	0.98 (0.92–1.05)	0.591
Moderate increase	0.90 (0.83–0.98)	0.021
Rapid increase	0.92 (0.85–1.00)	0.052

Note:

[a] In the CLHLS, the count of natural teeth was solely based on self-reporting, by the question: "How many natural teeth do you have (excluding dentures)?" If re-examined tooth count exceeded that of baseline tooth count, this phenomenon was identified as a "tooth increase". In general, the likelihood of new teeth emerging is significantly diminished in older adults. Consequently, participants with an increased number of re-examined teeth were excluded from the primary analysis (Figure 1), which also accounted for the possibility that some participants may have provided incorrect responses. Nevertheless, exceptional circumstances in which older adults may develop new teeth during re-examination were considered in the analytical process and treated as a sensitivity analysis.

In addition, for participants who had no teeth at baseline, it is logically reasonable to have an increase in tooth count during the follow-up, which differs from the assessment of tooth loss; consequently, those participants with a baseline tooth count of ≥0 were included in the assessment of tooth increase.

Based on the annual tooth increase, tooth increase progression was categorized into four groups: stable (annual rate: 0 teeth/year), slow increase (annual rate: >0, <2 teeth/year), moderate increase (annual rate: ≥2, <4 teeth/year), and rapid increase (annual rate: ≥4 teeth/year).

[b] With adjustment for sex, age, education, marital status, residence, co-residence, current smoking, current drinking, current regular exercise, regular intake of foods (fruit, vegetable, meat, fish, eggs, and beans), comorbidities (hypertension, diabetes, heart disease, cerebrovascular disease, and respiratory disease), ADL disability, denture, and baseline tooth count.

Abbreviations: ADL=activities of daily living, CI=confidence interval, HR=hazard ratio, CLHLS=Chinese Longitudinal Healthy Longevity Survey.

eTable 11. Incorporating participants with tooth increase, treating the data on tooth increase as missing, and using multiple imputation to assess the association between tooth loss progression and all-cause mortality[a] (n=12440)

	Adjusted HR (95% CI)[b]	p value
Stable	1.00 (ref)	
Slow loss	1.10 (1.00–1.21)	0.060
Moderate loss	1.18 (1.06–1.31)	0.003
Rapid loss	1.29 (1.13–1.47)	0.001

Note:

[a] For participants who had no teeth at baseline, it is logically reasonable to have an increase in tooth count during the follow-up, which differs from the assessment of tooth loss; consequently, those participants with a baseline tooth count of ≥0 were included in the assessment of tooth increase. A total of 4367 older participants with a baseline tooth count of ≥0 had both tooth increase and complete data. We defined the annual tooth increase as missing for these participants, then combined them with the 8073 study participants to form a comprehensive dataset. Finally, we performed multiple imputation by chained equations to create five datasets, of which the resultant model estimates for each were combined using Rubin`s rules.

[b] With adjustment for sex, age, education, marital status, residence, co-residence, current smoking, current drinking, current regular exercise, regular intake of foods (fruit, vegetable, meat, fish, eggs, and beans), comorbidities (hypertension, diabetes, heart disease, cerebrovascular disease, and respiratory disease), ADL disability, denture, and baseline tooth count.

Abbreviations: ADL=activities of daily living, CI=confidence interval, HR=hazard ratio.

eTable 12. Baseline characteristics of included participants and those with tooth increase

	All	Included participants	Participants with tooth increase (complete data)[a]	p value
No. of participants	12440	8073	4367	
Sex: male	5826 (46.8%)	3765 (46.6%)	2061 (47.2%)	0.564
Age (years)	83.0 (73.0–91.0)	83.0 (73.0–91.0)	83.0 (74.0–91.0)	0.028
Education				0.394
No school	7312 (58.8%)	4768 (59.1%)	2544 (58.3%)	
1 year or more	5128 (41.2%)	3305 (40.9%)	1823 (41.7%)	
Marital status				0.051
Not in marriage	7402 (59.5%)	4752 (58.9%)	2650 (60.7%)	
In marriage	5038 (40.5%)	3321 (41.1%)	1717 (39.3%)	
Residence				0.608
Urban	4748 (38.2%)	3095 (38.3%)	1653 (37.9%)	
Rural	7692 (61.8%)	4978 (61.7%)	2714 (62.1%)	
Co-residence				0.066
With family members	10416 (83.7%)	6789 (84.1%)	3627 (83.1%)	
Alone	1681 (13.5%)	1052 (13.0%)	629 (14.4%)	
In a nursing home	343 (2.8%)	232 (2.9%)	111 (2.5%)	
Current smoking	2735 (22.0%)	1744 (21.6%)	991 (22.7%)	0.168
Current drinking	2943 (23.7%)	1895 (23.5%)	1048 (24.0%)	0.525
Current regular exercise	4004 (32.2%)	2604 (32.3%)	1400 (32.1%)	0.838
Regular intake of foods				
Fruit	3642 (29.3%)	2382 (29.5%)	1260 (28.9%)	0.457
Vegetable	10750 (86.4%)	7007 (86.8%)	3743 (85.7%)	0.097
Meat	5168 (41.5%)	3364 (41.7%)	1804 (41.3%)	0.712
Fish	2901 (23.3%)	1839 (22.8%)	1062 (24.3%)	0.055
Eggs	5497 (44.2%)	3567 (44.2%)	1930 (44.2%)	1.000
Beans	4692 (37.7%)	3010 (37.3%)	1682 (38.5%)	0.182
Comorbidities				
Hypertension	2059 (16.6%)	1320 (16.4%)	739 (16.9%)	0.428
Diabetes	220 (1.8%)	146 (1.8%)	74 (1.7%)	0.697
Heart disease	933 (7.5%)	613 (7.6%)	320 (7.3%)	0.616
Cerebrovascular disease	491 (3.9%)	325 (4.0%)	166 (3.8%)	0.572
Respiratory disease	1280 (10.3%)	845 (10.5%)	435 (10.0%)	0.392
ADL disability	1718 (13.8%)	1080 (13.4%)	638 (14.6%)	0.061
Denture	2509 (20.2%)	1377 (17.1%)	1132 (25.9%)	<0.001
Baseline tooth count	8.0 (3.0–20.0)	11.0 (5.0–23.0)	5.0 (0.0–13.0)	<0.001
Re-examined tooth count	7.0 (2.0–18.0)	5.0 (1.0–12.0)	13.0 (6.0–24.0)	<0.001
Annual tooth loss (teeth/year)	1.6 (0.6–3.4)	1.4 (0.5–3.2)	1.9 (0.9–3.8)[b]	<0.001

Note:
Values are median (IQR) or n (%).
[a] For participants who had no teeth at baseline, it is logically reasonable to have an increase in tooth count during the follow-up, which differs from the assessment of tooth loss; consequently, those participants with a baseline tooth count of ≥0 were included in the assessment of tooth increase. A total of 4367 older participants with a baseline tooth count of ≥0 had both tooth increase and complete data.
[b] Indicating tooth increase.
Abbreviations: ADL=activities of daily living, IQR=inter-quartile range.

eFigure 1. Distributions of annual tooth loss

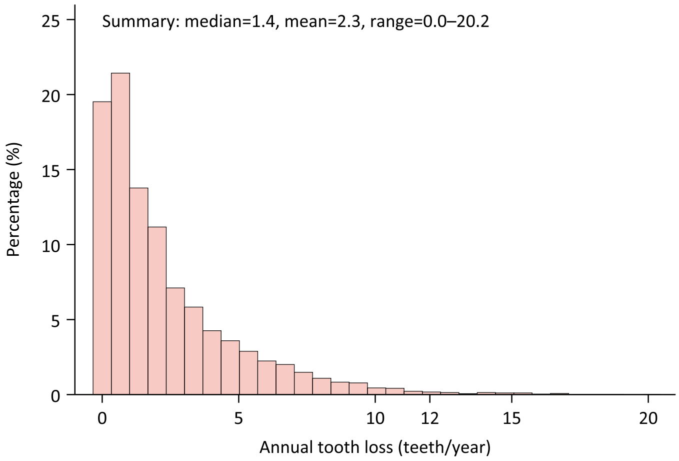

eFigure 2. Associations between baseline tooth count and re-examined tooth count and all-cause mortality

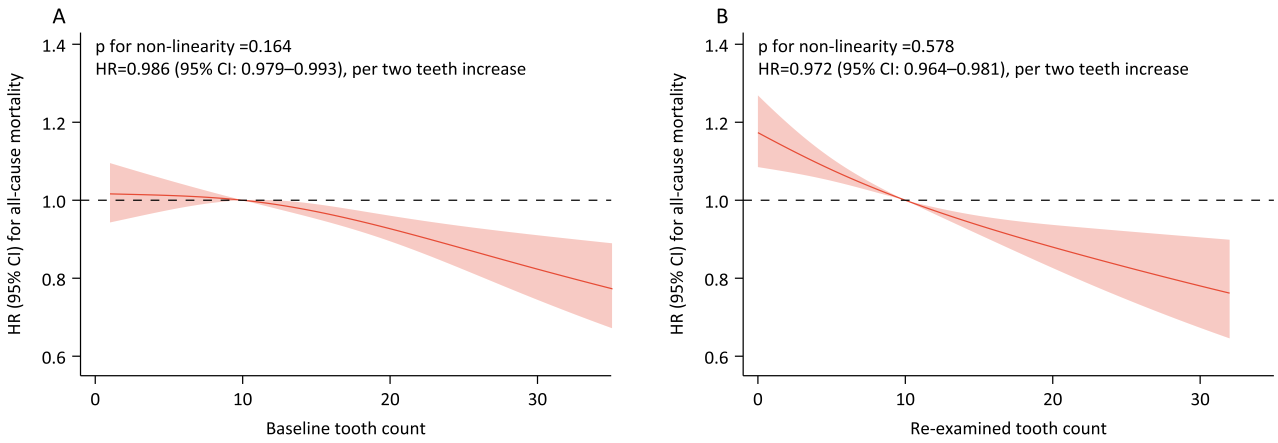

Note:
To examine the association between baseline tooth count (A) and re-examined tooth count (B) and all-cause mortality, we fitted a Cox regression model with a restricted cubic spline with three knots, with adjustment for sex, age, education, marital status, residence, co-residence, current smoking, current drinking, current regular exercise, regular intake of foods (fruit, vegetable, meat, fish, eggs, and beans), comorbidities (hypertension, diabetes, heart disease, cerebrovascular disease, and respiratory disease), ADL disability, and denture. The solid line represents the point estimates of HRs for mortality, while the shaded area indicates the corresponding 95% CIs.
Abbreviations: ADL=activities of daily living, CI=confidence interval, HR=hazard ratio.

eFigure 3. Stratified analyses by potential modifiers of the association between tooth loss progression and all-cause mortality (Figure 3 continued)

Tooth loss progression	Deaths/total (%)	Adjusted HR (95% CI)	Deaths/total (%)	Adjusted HR (95% CI)	p for interaction
	Co-residence: alone/in a nursing home		Co-residence: with family members		
Stable	140/207 (67.6%)	1.00 (ref)	703/1065 (66.0%)	1.00 (ref)	
Slow loss	386/562 (68.7%)	1.32 (1.08–1.60)	2019/3050 (66.2%)	1.07 (0.98–1.17)	0.335
Moderate loss	191/277 (69.0%)	1.36 (1.08–1.72)	826/1372 (60.2%)	1.17 (1.05–1.30)	
Rapid loss	163/238 (68.5%)	1.58 (1.20–2.09)	748/1302 (57.5%)	1.29 (1.15–1.45)	
	Lifestyle[a]: unhealthy		Lifestyle[a]: healthy		
Stable	679/1003 (67.7%)	1.00 (ref)	164/269 (61.0%)	1.00 (ref)	
Slow loss	1974/2917 (67.7%)	1.09 (1.00–1.19)	431/695 (62.0%)	1.18 (0.98–1.41)	0.792
Moderate loss	833/1327 (62.8%)	1.19 (1.07–1.33)	184/322 (57.1%)	1.19 (0.95–1.49)	
Rapid loss	741/1225 (60.5%)	1.31 (1.16–1.48)	170/315 (54.0%)	1.34 (1.04–1.72)	
	Comorbidities[b]: no		Comorbidities[b]: yes		
Stable	559/851 (65.7%)	1.00 (ref)	284/421 (67.5%)	1.00 (ref)	
Slow loss	1690/2477 (68.2%)	1.21 (1.09–1.33)	715/1135 (63.0%)	0.93 (0.81–1.07)	0.027
Moderate loss	719/1153 (62.4%)	1.28 (1.14–1.44)	298/496 (60.1%)	1.05 (0.89–1.25)	
Rapid loss	631/1068 (59.1%)	1.40 (1.23–1.60)	280/472 (59.3%)	1.21 (1.00–1.46)	
	ADL disability: no		ADL disability: yes		
Stable	673/1084 (62.1%)	1.00 (ref)	170/188 (90.4%)	1.00 (ref)	
Slow loss	1926/3065 (62.8%)	1.13 (1.03–1.23)	479/547 (87.6%)	1.05 (0.88–1.26)	0.912
Moderate loss	864/1468 (58.9%)	1.21 (1.09–1.35)	153/181 (84.5%)	1.13 (0.89–1.43)	
Rapid loss	773/1376 (56.2%)	1.34 (1.19–1.50)	138/164 (84.1%)	1.35 (1.02–1.80)	

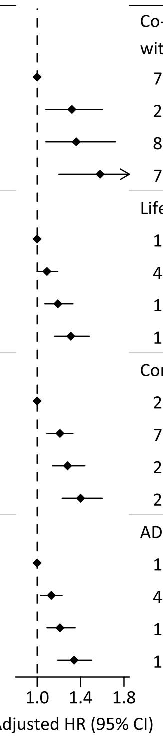

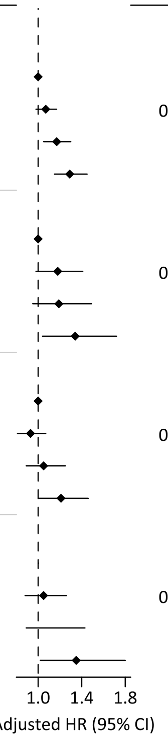

Note:

[a] For the lifestyle, a participant would be defined as "healthy" with meeting all the criterions, including current smoking (no), current drinking (no), current regular exercise (yes); otherwise, he/she was defined as "unhealthy".

[b] For comorbidities, any of the diseases shown in Table 1 was present, and the participant would be defined as "yes"; otherwise, he/she was defined as "no".

Each stratification was adjusted for all factors, including sex, age, education, marital status, residence, co-residence, current smoking, current drinking, current regular exercise, regular intake of foods (fruit, vegetable, meat, fish, eggs, and beans), comorbidities (hypertension, diabetes, heart disease, cerebrovascular disease, and respiratory disease), ADL disability, denture, and baseline tooth count; however, the stratification factor itself was excluded from the adjustment.

Abbreviations: ADL=activities of daily living, CI=confidence interval, HR=hazard ratio.
